# Hydrogeochemical evolution processes, groundwater quality, and non-carcinogenic risk assessment of nitrate-enriched groundwater to human health in different seasons in the Hawler (Erbil) and Bnaslawa Urbans, Iraq

**DOI:** 10.1007/s11356-024-32715-1

**Published:** 2024-03-18

**Authors:** Jawhar Mohammed-Shukur Tawfeeq, Erkan Dişli, Masoud Hussein Hamed

**Affiliations:** 1 Department of Environmental Engineering, Faculty of Engineering, Van Yüzüncü Yıl University, Van, 65080 Türkiye; 2 Department of Geology, College of Science, Salahaddin University, Erbil, 44001 Iraq

**Keywords:** Hydrogeochemistry, Groundwater elevation, Anthropogenic processes, Entropy water quality index, Non-carcinogenic risk, Erbil

## Abstract

The main objectives of this research are to assess groundwater, a primary source of drinking water in the urban areas of Hawler (Erbil) and Bnaslawa in northern Iraq, and the non-carcinogenic human health risks of nitrate contamination associated with drinking water quality. For this purpose, twenty-seven groundwater samples were collected from wells to assess the hydrogeochemical characteristics and groundwater quality for both natural and anthropogenic purposes during the wet (May 2020) and dry (September 2020) seasons. During the wet and dry seasons, NO_3_^−^ in groundwater ranged from 14.00 to 61.00 mg/L and 12.00 to 60.00 mg/L, with an average value of 35.70 and 29.00 mg/L, respectively. Approximately 25.92% of the samples exceeded the permissible limit of the WHO (2011) drinking water standard. The ratios of NO_3_^−^/Na^+^ vs. Cl^−^/Na^+^ and SO_4_^2−^/Na^+^ vs. NO_3_^−^/Na^+^ indicate the effect of agricultural activities and wastewater leaking from cesspools or septic tanks on the quality of groundwater during the wet and dry seasons. The entropy weighted water quality index method ranked 62.5% and 75% of the urban groundwater as not recommended for drinking, and the remaining samples are moderately suitable in both wet and dry seasons. The non-carcinogenic human health risk assessment displayed that during the wet and dry seasons, 29.6% and 25.9% of adults, 48% and 30% of children, and 48.1% and 29.6% of infants were exposed to increased concentrations of nitrate in groundwater. Due to high nitrate in drinking water, non-carcinogenic human health risk levels vary as infant > child > adults. The main findings obtained from this study can assist policymakers in better understanding the hydrogeochemical properties of groundwater in terms of drinking water safety, thereby facilitating the management of water resources to take the necessary measures.

## Introduction

Freshwater resources are known as one of the most vital living resources that are indispensable for the survival of every living thing in all habitat systems in various parts of the world (Dişli 2018a; Marghade 2020; Naik et al. 2022). Owing to their widespread availability and accessibility, these resources are used as the primary source of drinking water to ensure sustainable developing countries, especially in semi-arid and arid regions having rainfall scarcity or no rainfall. As a consequence, there is a reduction in the amount of fresh water resources, especially groundwater resources (Dişli 2018b; Öztürk and Dişli 2022; Xiao et al. 2022). This reliance on groundwater has increased uncontrollably over the past two decades, attributed to global climate changes, population growth, and, accordingly, significant development in the industrial and agricultural sectors (Adimalla et al. 2020). The easy access to groundwater resources due to quality, quantity, and technological development in recent years has been intrinsically linked to human health, economic development, and social prosperity, depending on the evolution or decline of human populations (He et al. 2019; Maskooni et al. 2020; Bretcan et al. 2022). Furthermore, it is recognized that having a groundwater resource that is safe to drink, renewable, and has economic value is one of the most significant key drivers of sustainable development for a nation today (Naik et al. 2022). Consequently, the increased demand for groundwater necessitates prompt and effective actions from relevant public and private organizations to protect its quality for human consumption and other purposes (Mahammad et al. 2022).

Groundwater resources are used as primarily natural freshwater resources within the hydrogeological system where they are located because they are less sensitive to physical and bacteriological deterioration than surface water resources (Li et al. 2023). Seasonal-spatial variations in groundwater quality are due to a variety of very complex geochemical and anthropogenic processes, such as mineral dissolution and precipitation, residence time, organic matter decomposition, aerobic respiration, nitrification and denitrification, oxidation-reduction, and ion exchange reactions (Dişli 2017; Sako et al. 2018; Li and Wu 2019; Kumar and Augustine 2022). However, in recent years, with changing land use/land cover patterns such as rapid and unplanned population growth, mining and high-intensity agricultural lands, swift industrial development, and open landfill sites, the groundwater environment has been seriously affected by pollutants from human activities (He et al. 2019; Dişli et al. 2021). Therefore, the widespread contamination of groundwater has led to a serious deterioration of its quality rather than its quantity (Aouiti et al. 2021). Recently, groundwater quality has been a significant factor as its quantity, because it plays an important role in deciding the adequacy of water demand for ever-increasing agricultural, industrial, and domestic water uses in connection with supply and demand imbalances for water resources (Dişli and Gülyüz 2020; Masood et al. 2022; Smail and Dişli 2023). As a result, the sustainability of the quality of groundwater globally is crucial for the health of consumers in the long term (Adimalla 2021; Amiri et al. 2022). Recent approaches in water resource management have predominantly focused on investigating the hydrogeochemical evolution and characterizing sources of pollution (Mia et al. 2023). Of these approaches, water quality indices (WQIs), multivariate statistical approaches, and analytical hierarchy process (AHP) methods have been used to reveal the hydrochemical properties of groundwater, control factors of chemical composition, and mechanisms affecting groundwater quality change processes (Rezaei et al. 2020). The water quality index (WQI), introduced by Horton (1965), is a numerical model used to estimate the entire chemical relationship between the water quality of surface water and/or groundwater resources (Panneerselvam et al. 2022; Mahammad et al. 2022). Groundwater quality assessment methods have gone through various stages from traditional WQI to entropy-weighted water quality index (EWQI), and EWQI has become the most widely used method today. EWQI, which uses entropy values including a large number of hydrochemical parameter variables, has been accepted as a more feasible and sensitive method due to its more comprehensive calculation and suitability for human consumption according to the groundwater quality standard (Wu et al. 2018; Singha et al. 2021; Jannat et al. 2022). In addition, EWQI, now integrated with GIS, is one of the effective methods used to summarize the composition and characteristics of groundwater (Panneerselvam et al. 2022).

The most common observed pollutant of anthropogenic origin in groundwater resources is nitrate (NO_3_^−^), and intensive fertilizer used in agricultural areas and infiltrate from cesspools are often associated with a high NO_3_^−^ level in groundwater. Long-dated consumption of water contaminated with high concentrations of NO_3_^−^ is known to cause reduced oxygen-carrying capacity of the blood in human blood, blue baby syndrome in infants, brain and spinal cord disease, and stomach cancer (Bretcan et al. 2022; Naik et al. 2022). Domestic and animal waste, as well as wastewater leaking from cesspools or septic tanks, polluted urban streams, improperly dumped solid wastes, and agricultural activities, contributes to pollution both in the surface and groundwater resources (El Baghdadi et al. 2015). Hence, the assessment of the sensitivity of groundwater to pollution, especially depending on the changed natural patterns of land cover/land use (LULC), has evolved into an important step in the sustainable development of urban areas (Karunanidhi et al. 2020). Recently, various techniques have been used to assess groundwater quality and decision-making around the world. These methods include the following: fuzzy logic model (Kamrani et al. 2016), a technique for order of preference by similarity to ideal solution (TOPSIS) (Gorgij et al. 2019), hierarchical analysis (Deng et al. 2017), set pair analysis (Tian and Wu 2019; Su et al. 2019; Lu et al. 2019), kriging model (Fallah et al. 2019), WQI (Chen et al. 2019), EWQI (Islam et al. 2017), the improved water quality index (Wang et al. 2018; Zhang et al. 2020a), extra tree regression model (Asadollah et al. 2021), and matter-element extension assessment method (Wang and Li 2022).

In developing countries such as Iraq, especially due to surface water source scarcity, groundwater is mostly used for irrigation activities and drinking purposes. The reliance on groundwater in the sub-basin area has increased tremendously over the past two decades, driven by substantial growth in the agricultural and industrial sectors as well as population expansion. This situation has also triggered the pollution of groundwater, especially due to the inadequate of infrastructure for settlements throughout the region. As a result, hydrogeochemical characterization, groundwater quality, and health risk assessments for drinking purposes are still lacking in many regions. Therefore, the aim of this study is (1) to describe the hydrogeochemical characterization of groundwater in the study area, (2) to research the groundwater quality for drinking and identify sources of groundwater pollutants as origins, (3) to discern the influence of both natural (geogenic) and human-induced (anthropogenic) processes on groundwater chemistry, (4) to evaluate groundwater quality by using the EWQI, and (5) to estimate human health risk caused by high nitrate in drinking water. This study provides useful information for improving drinking water supply, particularly in addressing potential health risks associated with groundwater quality. Additionally, the results may aid planners and policymakers in identifying the most effective groundwater management techniques for the study area.

## Study area

**Fig. 1:**
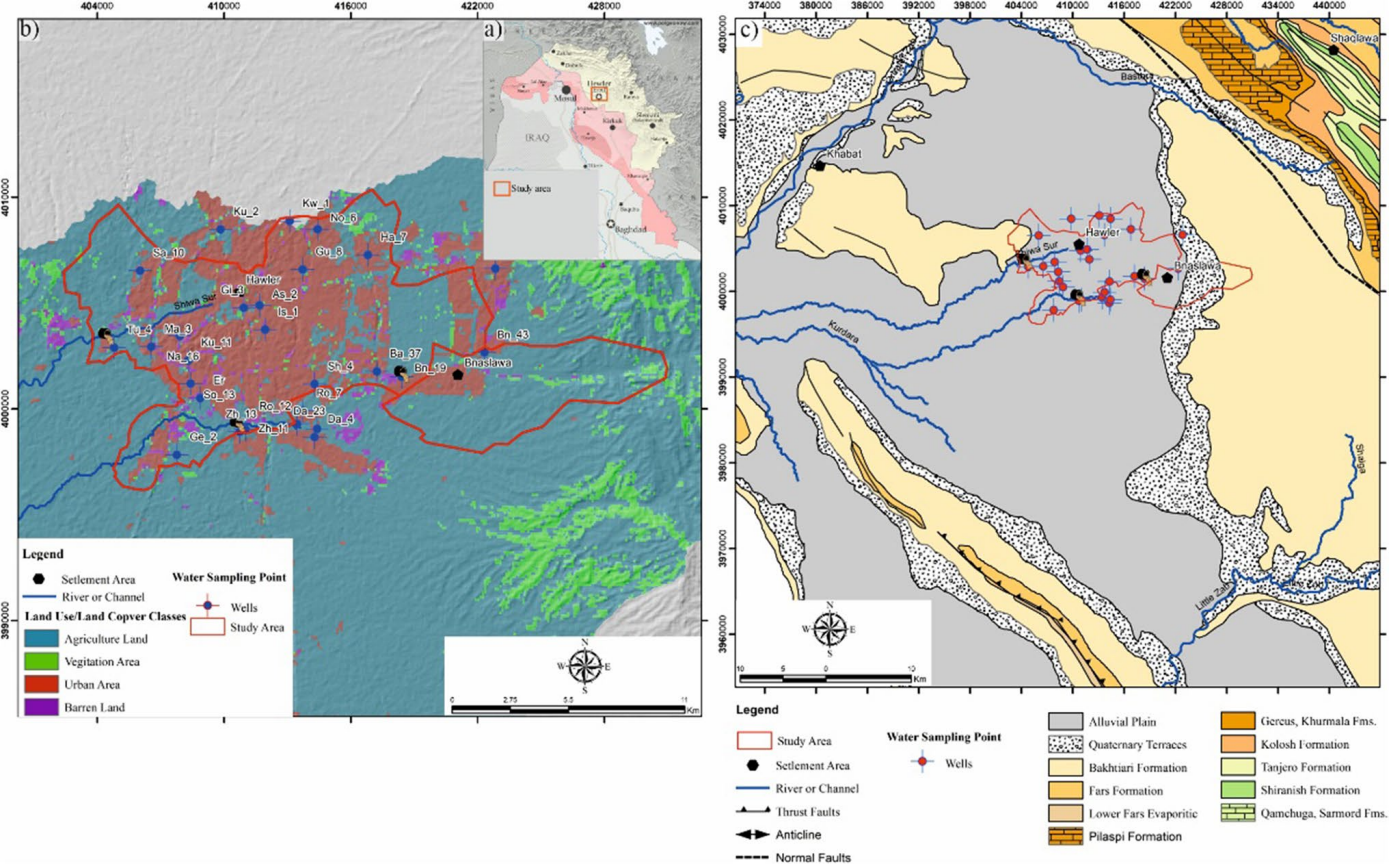
**a**, **b** Locations of the study area and **c** the geological map of the study area (**a** modified from Hameed et al. 2018; **c** modified from Le Garzic et al. 2019)

The Erbil province, located in the northeastern part of Iraq, is generally situated in highland and hilly regions where dry climates and water shortages are (Kareem et al. 2022). Geographically, it lies between east longitude 43°51′i20″–44°51′28″ and north latitude 36°08′30″–36°14′15″ covering about 15,074 km^2^ (Fig. 1a, b) (Rasul et al. 2018). According to the Hawler Meteorological Station in the city of Erbil, the average annual rainfall between 2000 and 2019 was 394.78 mm/year, the maximum rainfall in 2018 was 733.6 mm/year, and the minimum rainfall in 2010 was 260.4 mm/year. The highest average rainfall was in January (69.85 mm/month), and the lowest average rainfall was in August (0.06 mm/month). The maximum average temperature in Erbil city was 23.7 °C in 2017; the minimum average temperature was 20.19 °C in the year 2003 as measured. Summer is hot and dry, and winter is cold and snowy (Tawfeeq 2021; Masoud et al. 2023).

The stratigraphy of the area in which the lower basin boundaries are located is typical of the Taurus Zagros fold-thrust belt, which stretches more than 2000 km from Turkey to Southeastern Iran (Tavani et al. 2018). The geological units in the sub-basin area are mostly composed of the Bakhtiari formation (Bai Hassan) and Quaternary deposits (Fig. 1c). The Pliocene-aged Bakhtiari Formation consists of variable units of conglomerate (different colors and grain sizes), clay, sandstone, and gravel. This formation is the most permeable and porous unit and is regarded as one of the best water-bearing formations (Buday and Jassim 1987). Quaternary deposits outcrop on weathered basement rock, mostly recent terraces, and alluvium, comprising mainly a mixture of gravel, sand, silt, and clay (Jassim and Goff 2006). Quaternary deposits (alluvium and terraces) are mostly unconfined aquifers in the study region, while the Bakhtiari Formation typically features semi-confined to the confined aquifer (Dizayee 2010). Groundwater, which constitutes the main water source, is obtained from both shallow and deep aquifers, initially from shallow wells and later from deep wells, due to the long-term drought prevailing in the region in recent years. The groundwater level ranges from 302 to 447 m in the wet season and from 290 to 432 m in the dry season. Throughout both the wet and dry seasons, the direction of groundwater flow is from northeast to southwest (Tawfeeq 2021).

## Materials and methods

### Field sampling and physicochemical analysis

In this study, twenty-seven random groundwater samples (27 well) with well depths ranging from 160 to 350 m were sampled and analyzed in the study area during two different seasons, namely the wet and dry seasons, in May (2020) and September (2020), respectively. The place of the sampling points was recorded using a handheld GPS device, and then, the data obtained were transferred onto a satellite image using ArcGIS 10 software (Fig. 1). The samples were collected after water was pumped from the wells in sterile plastic containers with a capacity of 1000 mL for about 4–5 min. The samples were stored in the refrigerator at 4 °C for later use. Hydrogen ion concentration (pH), total dissolved solids (TDS), and electrical conductivity (EC) were measured in situ using a portable digital pH/EC/TDS meter, respectively. HCO_3_^−^, Cl^−^, Ca^2+^, Mg^2+^, and SO_4_^2−^ were analyzed in the laboratory using standard methods (Table 1).

**Table 1:**
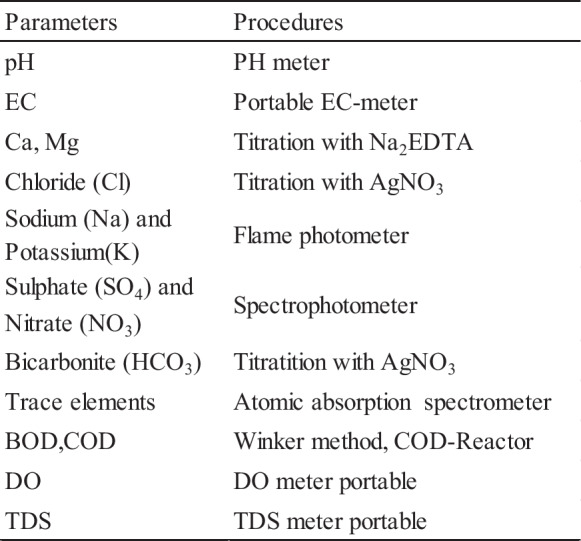
Analysis methods used in water quality parameter tests

## Methods

The hydrochemical composition and evolutionary processes of the groundwater samples in the study area were assessed using traditional methods, including Chadha’s diagram and ion ratio analysis. The groundwater quality and risks of contaminant loads on human health were carried out using an entropy-weighted water quality index and different health risk assessment models. In this study, the inverse distance-weighted (IDW) method from the spatial analysis tool included in ArcGIS 10.02 was used to examine the spatial-temporal change of groundwater quality parameters. IDW is indeed a widely used technique for spatial interpolation of data, including water quality parameters. It is commonly employed in geographic information systems (GISs) and environmental studies to estimate values at unsampled locations based on the values at nearby sampled locations (Panneerselvam et al. 2022).

### Groundwater quality models

#### Entropy weighted water quality index

The concept of information entropy, representing how stochastic an event can be, was initially formulated by Shannon (1948) and presented in the form of Shannon information entropy (Masood et al. 2022). Entropy serves as a robust method for quantifying the level of irregularity in a complex system and can provide a measure of the amount of useful information needed using the data obtained. Hence, entropy can be employed to assign weights to each parameter. When a notable discrepancy exists in a specific indicator between the evaluated parameters, the entropy tends to be smaller. This means that the weight of each parameter must be larger (Kamrani et al. 2016). The EWQI is a powerful method that takes into account all parameters measured for human intake/drinking needs and provides accurate and comprehensive information on the prevailing water quality in the process of assessing its suitability, high precision, and reliability for drinking purposes (Subba Rao et al. 2020; Dashora et al. 2022; Naik et al. 2022). The primary idea of EWQI is to define the weight of the assessment index based on the value of entropy. This weight represents it as a function of the numerical value of the overall groundwater quality status using the quality data of water in the study area (Li et al. 2021). The EWQI is used to combine hydrogeochemical data of groundwater resources into a representative value characterizing the quality of groundwater (Wu et al. 2018; Adimalla 2021). In this model, an initial evaluation matrix is first created, and then, the entropy weight is involved in each water quality parameter (Kumar and Augustine 2022). The algorithms developed to calculate entropy, entropy weight, and EWQI are based on the following five steps (Adimalla et al. 2020):

Step 1: The eigenvalue matrix *X* consists of an “*m*” total number of water samples monitored to evaluate the water quality and an “*n*” number of measured/analyzed hydrogeochemical parameters (Su et al. 2019; Wu et al. 2018), and then according to the observed data, hydrogeochemical parameters for each groundwater sample in the X eigenvalue matrix can be generated using Eq. 1:

1 $$X=\left[\begin{array}{cccc}{x}_{11}& {x}_{12}& .& {x}_{1n}\\ {}{x}_{21}& {x}_{22}& .& {x}_{2n}\\ {}.& .& .& .\\ {}{x}_{m1}& {x}_{m2}& .& {x}_{mn}\end{array}\right]$$

Step 2: In view of the fact that groundwater quality parameters have different units, the data from the first procedure have to be standardized to eliminate the effects of size and dimensions with the normalization function (Eq. 2) after transformation; standard evaluation matrix *Y* can be developed and shown below (Eq. 3):

2 $${y}_{ij}=\frac{x_{ij}-{\left({x}_{ij}\right)}_{min}}{{\left({x}_{ij}\right)}_{max}-{\left({x}_{ij}\right)}_{min}}$$

3 $$Y=\left[\begin{array}{cccc}{y}_{11}& {y}_{12}& .& {y}_{1n}\\ {}{y}_{21}& {y}_{22}& .& {y}_{2n}\\ {}.& .& .& .\\ {}{y}_{m1}& {y}_{m2}& .& {y}_{mn}\end{array}\right]$$

where *y*_*ij*_ is the normalized matrix, *x*_*ij*_ represents the value of hydrogeochemical parameter *j* of water sample *i*, and (*x*_*ij*_*)*_*min*_ and (*x*_*ij*_*)*_*max*_ represent the minimum and maximum values of the hydrogeochemical parameters in matrix *X*, respectively.

The parameter index amount *j* index in the *i* sample can be determined using Eq. 4 given below.

4 $${P}_{ij}={~}^{{y}_{ij}}\!\left/ \!{~}_{\sum_{i=1}^m{y}_{ij}}\right.$$

Step 3: The entropy value of the *j*th hydrogeochemical parameter, entropy *e*_*j*_, can be computed by using Eq. 5.

5 $${e}_j=-\frac{1}{lnm}\sum_{i=1}^m{P}_{ij}\mathit{\ln}{P}_{ij}$$

The smaller the quantity of entropy, the greater is the effectiveness of the parameter *j* index. In the formula, the entropy weight of *j* for each parameter *ω*_*j*_ is given in Eq. 6.

6 $${\omega}_j=\frac{1-{\mathcal{e}}_j}{\sum_{j=1}^n\left(1-{\mathcal{e}}_j\right)}$$

In step 4, the quality rating scale determination, *q*_*j*_, for each parameter *j* can be determined by Eq. 7:

7 $$\left\{\begin{array}{c}{q}_j=\frac{C_j}{S_j}x100\\ {}{q}_{pH}=\frac{C_{pH}-7}{S_{pH}-7}x100\end{array}\right.$$

where *c*_*j*_ is the concentration of chemical parameter “*j*” (mg/L) and *s*_*j*_ is the permissible limit of drinking water quality by national or international standards of the parameter concentration *j* (mg/L), respectively. *C*_pH_ and *S*_pH_ represent the value of pH and the permissible limit value of pH (6.5–8.5); if the pH value is greater than 7, measured in situ, *S*_pH_ should be considered as 8.5, while if the pH is less than 7, to verify that the value of “*q*_*j*_” is positive, the “*S*_pH_” should be equal to 6.5 (Adimalla et al. 2020). In the present study, the WHO (2011) guidelines for the drinking water quality standard have been used. The final step, step 5, of EWQI calculation will be (Eq. 8):

8 $$EWQI=\sum_{\mathrm{j}=1}^{\mathrm{n}}{\omega}_j{q}_j$$

Li et al. (2010) divided groundwater quality based on EWQI for drinking purposes into five different ranks (Table 2).

**Table 2:**
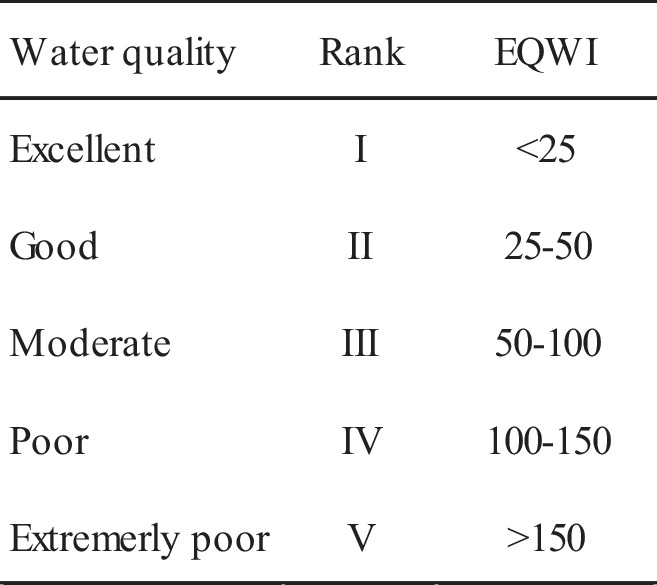
Groundwater quality classification standards based on EWQI for drinking purposes (Adimalla et al. 2020; Gao et al. 2022)

### Human health risk assessment modeling

Health risk assessment is a quantitative method used to assess the effects and risks of pollutant loads on the health of a human through exposure to drinking (oral consumption) and skin contact (dermal exposure) (USEPA 1989, 2004; He and Wu 2019; Marghade 2020; Adimalla et al. 2020; Asad et al. 2023). This revolutionary model, developed by the USEPA (U.S. Environmental Protection Agency), applies to the direct drinking of nitrate-containing contaminated water (Adimalla et al. 2020). The health risk assessment for people, both children and adult populations, was calculated using daily intake indices (CDI) and hazard coefficient (HQ). The receptors were divided into three different categories: i.e., infants (< 1 year of age), children (between 10 and 20 years of age), and adults (> 21 and < 64 years of age) (He and Wu 2019; Adimalla et al. 2020).

The CDI was calculated as follows (Eq. 9):

9 $$\mathrm{CDI}=\frac{C_w xIRWxEFxED}{BWxAT}$$

where CDI is the estimated chronic daily intake dose via drinking water for contaminants (mg/kg/day); *C*_*w*_ is the concentration of a notable contaminant in water in mg/L (nitrate 14 to 61 mg/L and 12 to 60 mg/L during the wet and dry season). IRW is the human intake rate of drinking water (L/day), and EF and ED denote the average exposure frequency (days/year) and the duration of exposure (years), respectively. BW represents the average body weight (kg), and AT is the average exposure time (days). The hazard quotient (HQ) and calculated potential hazard index (HI) by the drinking water intake pathway are computed as follows (USEPA 1989) (Eqs. 10 and 11):

10 $$\mathrm{HQ}=\frac{\mathrm{CDI}}{\mathrm{RfD}}$$

11 $$\mathrm{HI}=\sum \mathrm{HQ}$$

The non-carcinogenic reference dose (RfD) is 1.6 mg/kg/day for nitrate (USEPA 2019; Adimalla et al. 2020; Naik et al. 2022). According to USEPA (1989) and Ezugwu et al. (2019), the HI (non-carcinogenic risk) is classified based on the following categories: negligible (HI < 0.1), low risk (0.1 ≤ HI < 1), medium risk (1 ≤ HI < 4), and high risk (HI ≥ 4).

## Results and discussion

### Chemical compositions of groundwater

**Fig. 2:**
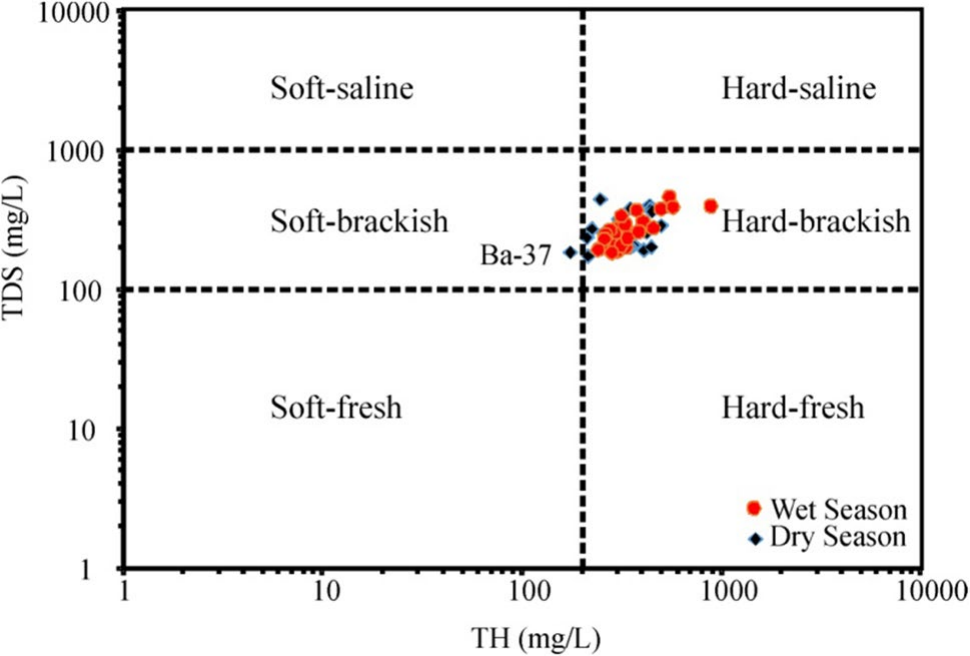
Scatter plots of TDS vs. TH indicating the quality of groundwater

The concentrations and summary statistics of the hydrochemical parameters were compared with the drinking water standards prescribed by WHO (2011) for the April (wet season) and September (dry season) 2020 seasons that were compiled in Table 3, respectively (Tawfeeq 2021). pH, a crucial parameter indicative of drinking water suitability, causes the water to corrode if it is low value in water resources, while very high pH values change the taste of water and adversely affect human skin and eye health (Wang and Li 2022). In the well water samples, the groundwater exhibited alkaline water quality, with pH ranging from 7.10 to 8.00 during the wet season and 7.10 to 7.90 during the dry season. The average pH values were 7.53 and 7.38 for the wet and dry seasons, respectively. Importantly, all groundwater samples were in the limit range of WHO (2011) drinking water quality standards (6.5–8.5). Alkalinity in most natural waters is due mainly to the presence of carbonate (CO_3_^2−^), bicarbonate (HCO_3_^−^), and hydroxyl (OH^−^) anions (Sajil Kumar et al. 2013). Groundwater samples had TDS values ranging from 183.00 to 459.00 mg/L and 173.00 to 441.00 mg/L, and their average values were 267.19 mg/L and 262.67 mg/L, respectively, during the wet and dry seasons. The EC values of groundwater samples were found in the range from 366.00 to 917.00 μS/cm and 346.00 to 882.00 μS/cm with an average of 533.81 and 524.96 μS/cm, respectively, during the wet and dry seasons. All the groundwater samples have TDS and EC values below the permissible limit of drinking water quality standards of 1000 mg/L and 1500 μS/cm suggested by WHO (2011). The considerable variation observed in the range of TDS and EC parameters in groundwater samples suggests a significant deviation in the concentration of ions. This variance can be attributed to natural processes such as soil-water/water-rock interaction as well as human activities including domestic effluents, irrigation return flow, and the use of fertilizer chemicals (Adimalla et al. 2020; Wang and Li 2022). The results indicate that total hardness (TH) (Dişli 2017) ranged between 240.17 and 882.87 mg/L during the wet season, with an average value of 359.29 mg/L. Notably, 88.89% of well water samples fell within the permissible limits established by WHO (2011) at 500 mg/L, except for three wells (Sa-10, Gl-3, and As-2). During the dry season, TH varied in the range of 174.282–497.48 mg/L, with an average value of 320.05 mg/L, found in the groundwater samples representing 100% of the samples falling within the permissible limit. Elevated levels of TH in groundwater resources generate an unpleasant in the water; abnormal concentrations when used continuously may also induce the formation of kidney stones and contribute to cardiovascular diseases in individuals (Chaudhary and Satheeshkumar 2018; Kumar and Augustine 2022). According to the plot TH vs TDS (Fig. 2), 26 out of 27 groundwater samples in the research area during the dry season were classified as the hard brackish type and the remaining (Ba-37) were soft brackish water type, while during the wet season, all groundwater samples were of hard brackish water type.

**Table 3:**
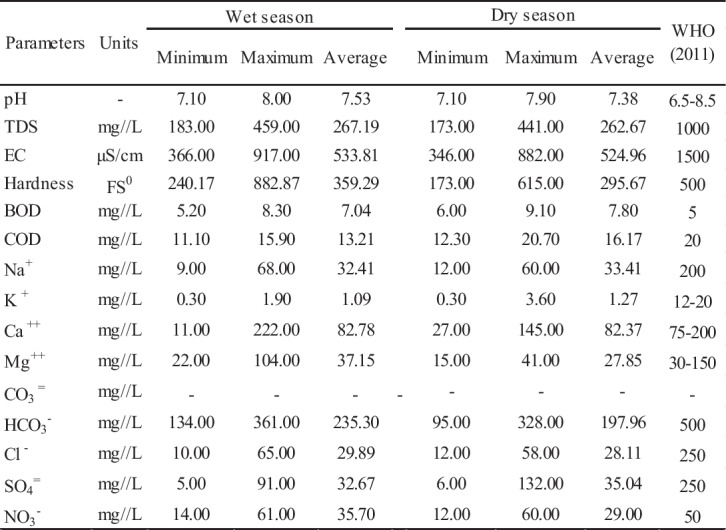
Statistical summary of physicochemical parameters of groundwater in the study area and drinking water standard

The parameters of biochemical oxygen demand (BOD) and chemical oxygen demand (COD) represent the organic pollution load in water resources (Barakat et al. 2016). During the wet season, the values of BOD and COD in the groundwater samples were found to vary from 5.20 to 8.30 and 11.10 to 15.90 mg/L, with an average value of 7.04 and 13.21 mg/L, respectively. However, during the dry season, the BOD and COD values ranged between 6.00 and 9.10 mg/L and 12.30 and 20.70 mg/L with an average value of 7.80 and 16.17 mg/L, respectively (Table 3). Especially, the BOD parameter in the groundwater samples may be associated with the leaching and transport of natural sewage from the cesspool, the wastewater channels, the municipal solid waste landfill sites, and agricultural and industrial pollution in the study area. In addition to these processes, the source of these parameters in wastewater channels is generally due to the untreated wastewater discharge from industrial areas and surface drainage of chemicals used in agricultural areas (Tawfeeq 2021). In both seasons, the concentration of BOD in eight samples is over the permissible limit for drinking water of 5 mg/L (WHO 2011). During the wet season, the COD concentrations of all groundwater samples remain within the range of 20 mg/L, while in the dry season, two well water samples are higher, exceeding the maximum permissible limit for drinking water (WHO 2011). The high BOD and COD in groundwater samples during the dry season demonstrate that rainwater is the primary source of recharge of the aquifer system and that the contamination capacity of groundwater is higher in the dry season than in the wet season (Wang and Li 2022).

According to the average value of chemical concentrations (in mg/L), the main anions and cations in the groundwater samples Ca^2+^ and HCO_3_^−^ and cation abundance followed the order Ca^2+^ > Mg^2+^ > Na^+^ > K^+^ and Ca^2+^ > Na^+^ > Mg^2+^ > K^+^ while the anion abundance followed the order HCO_3_^−^ > NO_3_^−^ > SO_4_^2−^ > Cl^−^ and HCO_3_^−^ > SO_4_^2−^ > NO_3_^−^ > Cl^−^ during the wet and dry seasons. The dominant cation of groundwater is Mg^+2^, and its content ranges from 22.00 to 104.00 mg/L and 15.00 to 41.00 mg/L, with average values of 37.15 mg/L and 27.85 mg/L in the wet season and the dry season, respectively. Ca^2+^ is the second predominant cation in the groundwater, and its concentration showed a wide range from 11.00 to 222.00 mg/L and 27.00 to 145.00 mg/L, with average values of 82.78 and 82.37 mg/L during the wet and dry seasons, respectively. Na^+^ and K^+^ were calculated within a range of 9.00–68.00 mg/L and 12.00–60.00 mg/L, with average values of 32.41 and 1.09 mg/L, and 0.30–1.90 mg/L and 0.30–3.06 mg/L with average values of 33.41 and 1.27 mg/L in the dry and wet seasons. In terms of major cations, considering the permissible limit of 200 mg/L, only Ca^2+^ demonstrates high concentrations in 3.7% of the samples (WHO 2011). HCO_3_^−^ and SO_4_^2−^ have been observed as first and second dominant anions ranging from 134.00 to 361.00 mg/L and 5.00 to 91.00 mg/L with average values of 235.00 and 32.67 mg/L in the wet season, and from 95.00 to 328.00 mg/L and 6.00 to132.00 mg/L with average values of 197.00 and 35.04 mg/L in the dry season. Cl^−^ fell in the range of 10.00–65.00 mg/L in the wet season and 12.00–58.00 mg/L in the dry season, with average values of 29.89 and 28.11 mg/L, respectively. The values of HCO_3_^−^, SO_4_^2−^, and Cl^−^ parameters were within the permissible limit of WHO (2011) for all seasons (Table 3). The abundance of these cations and anions varies from the process of mineralization and depending on anthropogenic sources, and the contact time between the rock-water and groundwater flow direction (Zhang et al. 2021).

### Chadha’s diagram

The hydrochemical facies of groundwater is always associated with the geological units of that region, and the distribution of the facies types is influenced by the geochemical reactions occurring within those geological units. Hydrogeochemical facies is used to classify based on the prevailing ions by using the Chadha (1999) rectangular diagram (Mao et al. 2021). The Chadha diagram is constructed by plotting the variance in milliequivalent percentages between alkaline earth ions (Ca^2+^ + Mg^2+^) and alkali metal ions (Na^+^ + K^+^) for cations on the *X*-axis. Simultaneously, the difference between CO_3_^2−^ + HCO_3_^−^ (acidic anions) and SO^2−^_4_ + Cl^−^ (strong acidic anions) for anions are marked on *Y*-axis, respectively (Ravikumar and Somashekar 2017). To describe the main properties of water, the rectangular area is separated into eight sub-areas, each representing a particular type of water, as in the Piper diagram (Piper 1944), and each rectangular area describes the overall water feature (Mao et al. 2021).

**Fig. 3:**
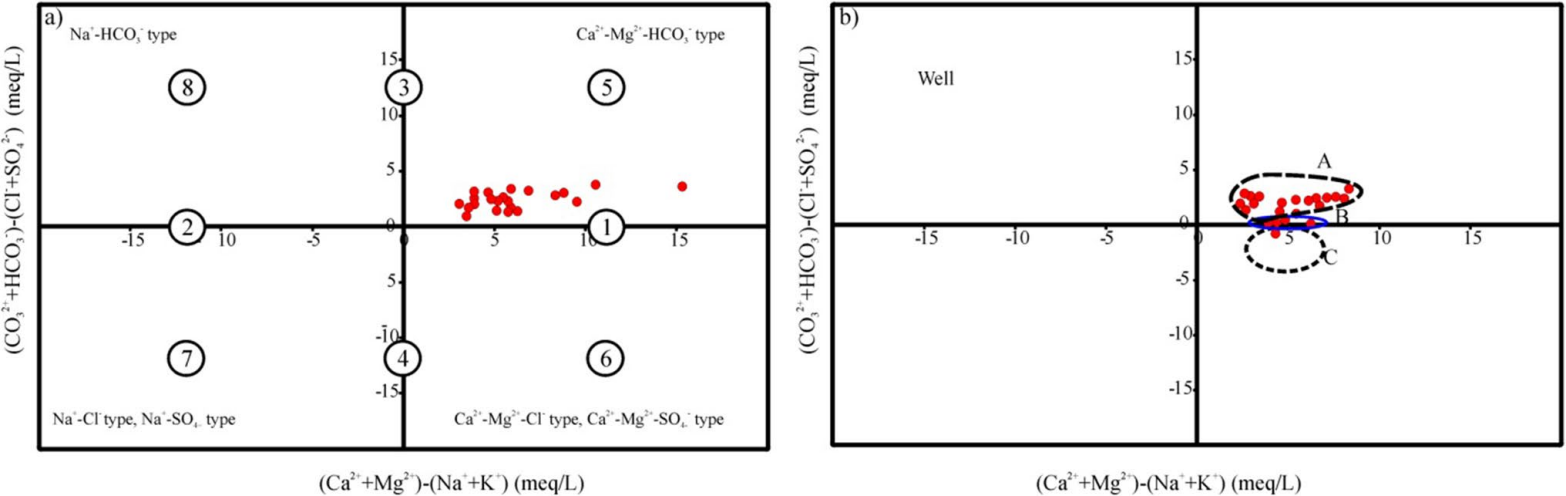
Water chemical facies: **a** wet season and **b** dry season

The plot of sub-fields on this diagram is shown in Fig. 3. The Chadha plot of the wet season displays that all of the groundwater samples occur in zone 5 (Ca-Mg-HCO_3_ water type; HCO_3_^−^dominant in Ca^2+^-Mg^2+^ type) with temporary hardness and Ca^2+^ + Mg^2+^ exceed Na^+^ + K^+^ (Fig. 3a). In contrast, during the dry season (Fig. 3b), the plot shows that group A (81.48%) mainly in zone 5 falls under the field of Ca^2+^-Mg^2+^-HCO_3_^−^ water type with temporary hardness, whereas group B (14.81%) in zone 1 falls on the alkaline earth metals that exceed alkali metals (Ca + Mg > Na + K). The remaining samples (group C; 3.70%) in zone 6 belong to the subfield of Ca^2+^-Mg^2+^-Cl^−^ water type representing the dominance of reverse ion exchange in these samples; this type of water displays a permanent hardness. This type of water can be recognized as the first step of rock-water interactions occurring in diluted solutions due to the affluence of carbonate lithologies in the study area.

### Identification of geochemical signatures

Ionic molar ratio graphs of ion concentrations are frequently employed as geochemical signatures, providing insights into the impact of land use/land cover patterns, natural influences, and anthropogenic inputs on groundwater hydrogeochemistry. These graphs showcase the molar ratios of ion concentrations derived from field measurements (Sajil Kumar 2014; Marghade 2020). One of the ionic ratios used in the process of evaluating the effect of rock weathering or evaporation process on groundwater chemistry due to water-rock interaction is Na^+^/Cl^−^ (Meybeck 1987; Dişli 2018b; Sunkari et al. 2022). In groundwater resources, Na^+^ is attributed to the dissolution of halite through natural processes and the decomposition of minerals containing sodium, both resulting from chemical reactions occurring during the water-rock interaction (Mao et al. 2021). If the Na^+^/Cl^−^ ratio is approximately 1, the main process that controls ion content is halite dissolution. Moreover, if the Na^+^/Cl^−^ molar ratios are higher than 1, then sodium may result from Na^+^ released into groundwater during the Na^+^-rich silicate weathering and by ion exchange reactions that involve the replacement of Ca^2+^ bound to newly formed clay mineral surface (Zhang et al. 2020b; Mao et al. 2021; Öztürk and Dişli 2022; Sunkari et al. 2022) (Eq. 12).

12 $$2{\mathrm{Na}\mathrm{AlSiO}}_3\mathrm{O}+{\mathrm{Ca}}^{2+}\leftrightarrow {\mathrm{Ca}\mathrm{AlSiO}}_3\mathrm{O}+2{\mathrm{Na}}^{+}$$

**Fig. 4:**
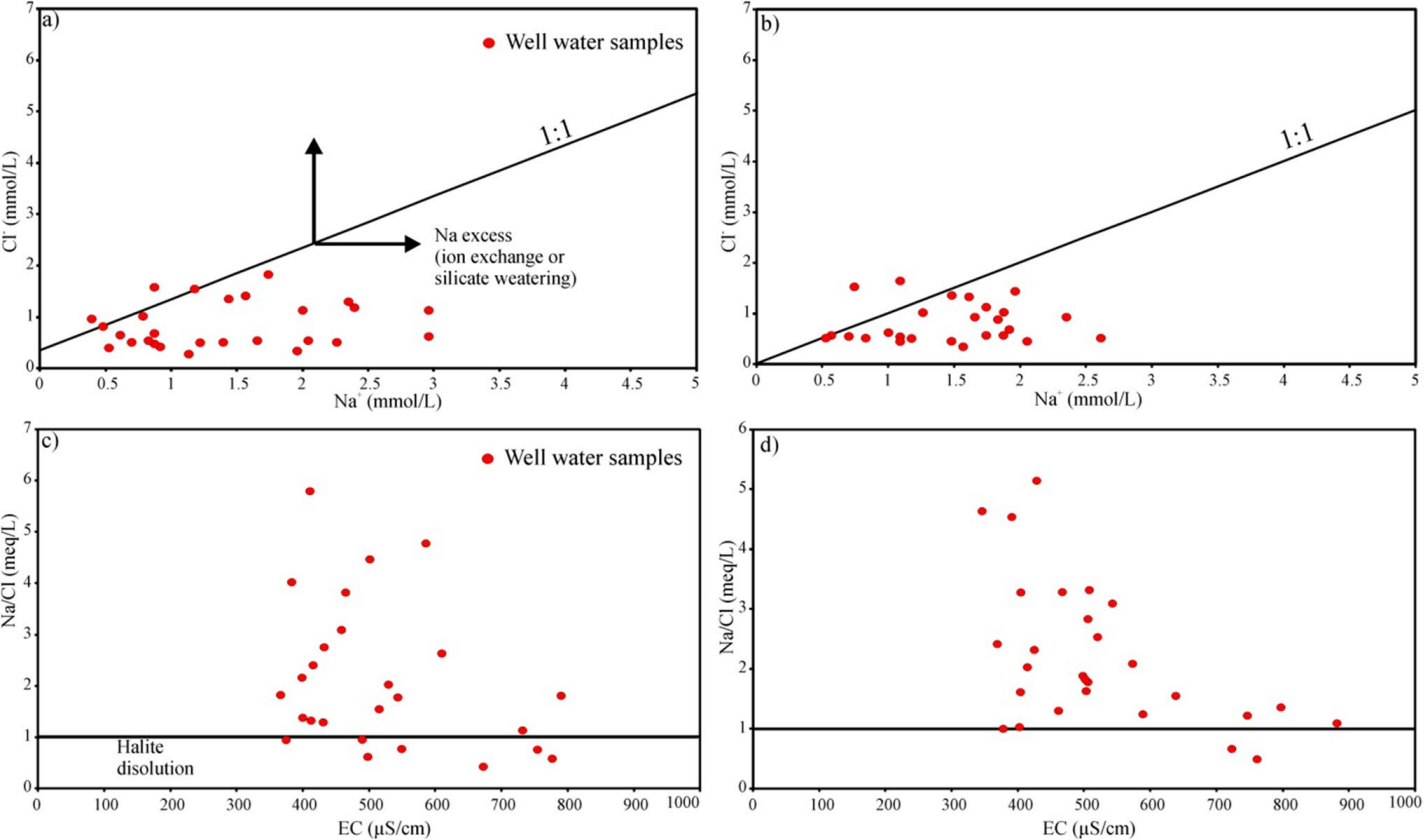
The plots show the mutual relationship between **a**, **b** Cl versus Na and **c**, **d** Na/Cl versus EC in the well water samples in the research area during the wet and dry seasons

During the wet and dry seasons, the range ratio of Na^+^/Cl^−^ in groundwater samples increased from 0.41 to 5.78 and from 0.49 to 5.14, with an average value of 2.07 and 2.19, and in most groundwater samples, the Na^+^/Cl^−^ ratio was greater than 1 (Fig. 4a, b). The bivariate diagram between Na^+^ against Cl^−^ (Fig. 4a, b) suggests that the groundwater samples generally have excess sodium content during the wet and dry seasons, and 74.07% and 92.59% of the groundwater samples fall below the (1:1) molar line. Figure 4a, b also confirm that ion exchange reactions and silicate weathering are the dominant sources of Na^+^ enrichment in the aquifer system, especially in the dry season and the anion HCO_3_^−^ that usually dominates in such groundwater (Samtio et al. 2023). Accordingly, as a result of the reaction of silicate minerals such as albite with weak carbonic acid in groundwater flow systems, HCO_3_^−^ is usually released as the end product (Eq. 13) (Sunkari et al. 2022). The similar situation above is also supported by the results from the EC vs. Na^+^/Cl^−^ diagram (Fig. 4c, d). As can be seen in Fig. 4c, d, the Na^+^/Cl^−^ molar ratio at 25.93% and 7.41% of the well water samples during the wet and dry periods is lower than 1, indicating greater mobility of Cl^−^ ions than Na^+^, and these show that the impact of anthropogenic input or inverse ion exchange on groundwater is predominant. The lack of any halite-containing geological units throughout the Erbil-Central sub-basin, which includes the study area, shows the contribution of anthropogenic sources to the Cl^−^ions in the groundwater released from the halite (Marghade 2020).

13 $$2{\mathrm{Na}\mathrm{AlSi}}_3{\mathrm{O}}_8+2{\mathrm{CO}}_2+11{\mathrm{H}}_2\mathrm{O}\leftrightarrow {\mathrm{Al}}_2\mathrm{SiO}5{\left(\mathrm{OH}\right)}_4+2{\mathrm{Na}}^{+}+2{\mathrm{H}\mathrm{CO}}_{3^{-}}+4{\mathrm{H}}_4{\mathrm{SiO}}_4$$

**Fig. 5:**
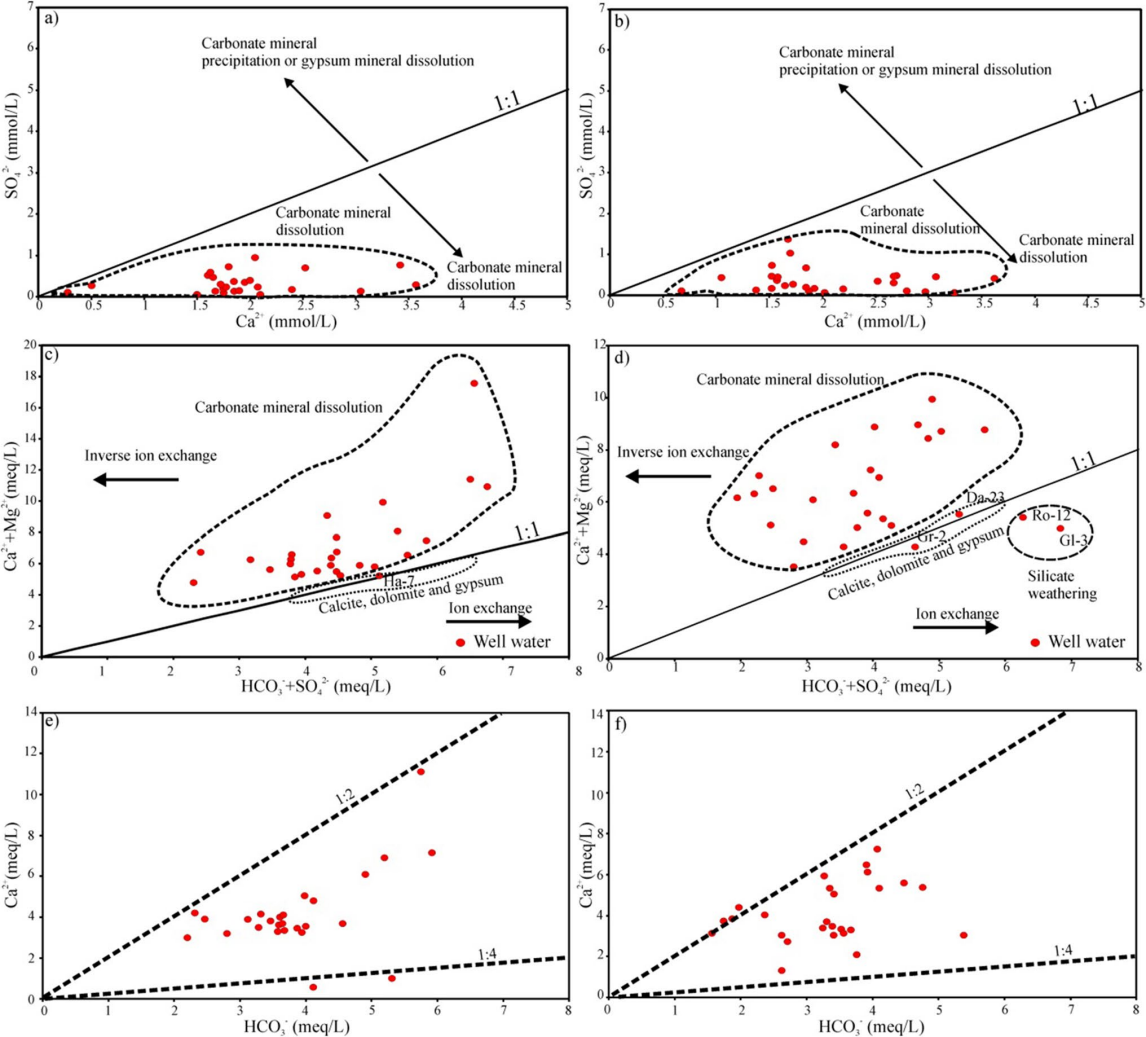
Relationships between major ion concentrations in the groundwater **a**, **b** SO_4_^2−^ against Ca^2+^, **c**, **d** Ca^2+^ + Mg^2+^ against SO_4_^2−^ + HCO_3_^−^, and **e**, **f** Ca^2+^against HCO_3_^−^ during the wet and dry season

The Ca^2+^ and SO_4_^2−^ bivariate plot demonstrates that during wet and dry seasons (Fig. 5a, b) all of the well water samples are situated below the 1:1 aquiline, showing an excess in Ca^2+^, suggesting that the carbonate mineral dissolution was dominant in the hydrogeochemical evolution of groundwater in the study area and dissolution of silicate minerals was a secondary process (Aouiti et al. 2021). The other important hydrogeochemical ratio, indicative of carbonate (calcite, CaCO_3_ (Eq. 14); dolomite, CaMg(CO_3_)_2_) dissolution (Eq. 15), silicate weathering (Eq. 16), and gypsum or anhydrite CaSO_4_ (Eq. 17) is due to the interrelationship of the alkaline earth metals (Ca^2+^ + Mg^2+^) with bicarbonate and sulfate (HCO_3_^−^ + SO_4_^2−^) (Appelo and Postma 2005; Dişli 2018b; Marghade 2020; Gao et al. 2022). Groundwater samples are scattered at the left (96.30 and 85.19%) and right (3.7 and 14.81%) part of the 1:1 (line) line in the wet and dry seasons (Fig. 5c, d). The scatter diagram of Ca^2+^ + Mg^2+^ versus HCO_3_^−^ + SO_4_^2−^ indicates a higher number of well water samples above the 1:1 theoretical line during the wet and dry periods indicating that carbonate dissolution dominates the hydrogeochemical process (Adimalla et al. 2020; Dişli and Gülyüz 2020). Moreover, the three samples (well Ha-7 during the wet season, wells Gr-2 and Da-23 during the dry season) falling close to the equiline of 1:1 show that the dissolution of carbonate and evaporate minerals also affected the hydrogeochemical characteristics. In addition, the two well water samples (Ro-12 and Gl-3) exhibiting values below the theoretical line of 1:1 during the dry season indicate that silicate weathering has influenced the geochemical characteristics of groundwater. In the scatter plot of Ca^2+^ and HCO_3_^−^, approximately 92.6% and 85.2% of well water samples during the dry and wet seasons, respectively, fall between the ratios of 1:2 and 1:4. This suggests that calcite dissolution, driven by carbonic acid alone, is a dominant factor influencing the groundwater chemistry in these locations (Fig. 5e, f).

The dissolution of calcite is

14 $${\mathrm{Ca}\mathrm{CO}}_3+{\mathrm{H}}_2{\mathrm{CO}}_3\leftrightarrow {\mathrm{Ca}}^{2+}+2{\mathrm{H}\mathrm{CO}}_{3^{-}}$$

Dissolution of dolomite is

15 $${\mathrm{Ca}}_{\mathrm{x}}{\mathrm{Mg}}_{\mathrm{l}-\mathrm{x}}{\mathrm{CO}}_3+{\mathrm{H}}_2{\mathrm{CO}}_3\leftrightarrow {\mathrm{Ca}}^{2+}+\left(1-\mathrm{x}\right){\mathrm{Mg}}^{2+}+2{\mathrm{H}\mathrm{CO}}_{3^{-}}$$

The weathering of silicate minerals is

16 $$\mathrm{Ca}{\mathrm{Al}}_2{\mathrm{Si}}_2{\mathrm{O}}_8+{\mathrm{H}}_2{\mathrm{CO}}_3+{~}^{1}\!\left/ \!{~}_{2}\right.{\mathrm{O}}_2\to {\mathrm{Al}}_2{\mathrm{Si}}_2{\mathrm{O}}_5{\left(\mathrm{OH}\right)}_4+{\mathrm{Ca}}^{2+}+{\mathrm{CO}}_3^{2-}$$

(Plagioclase)

$$\mathrm{CaMg}\left({\mathrm{Si}}_2{\mathrm{O}}_6\right)+4{\mathrm{CO}}_2+6{\mathrm{H}}_2\mathrm{O}\leftrightarrow {\mathrm{Ca}}^{2+}+{\mathrm{Mg}}^{2+}+4{\mathrm{H}\mathrm{CO}}_{3^{-}}+2\mathrm{Si}{\left(\mathrm{OH}\right)}_4(6)$$

(Pyroxenes)

The dissolution of evaporate is

17 $${\mathrm{Ca}\mathrm{SO}}_4+\leftrightarrow {\mathrm{Ca}}^{2+}+{\mathrm{SO}}_{4^{2-}}$$

(Gypsum)

$${\mathrm{Ca}\mathrm{SO}}_4\cdot 2{\mathrm{H}}_2\mathrm{O}\to {\mathrm{Ca}}^{2+}+{\mathrm{SO}}_{4^{2-}}+2{\mathrm{H}}_2\mathrm{O}$$

(Anhydrite)

**Fig. 6:**
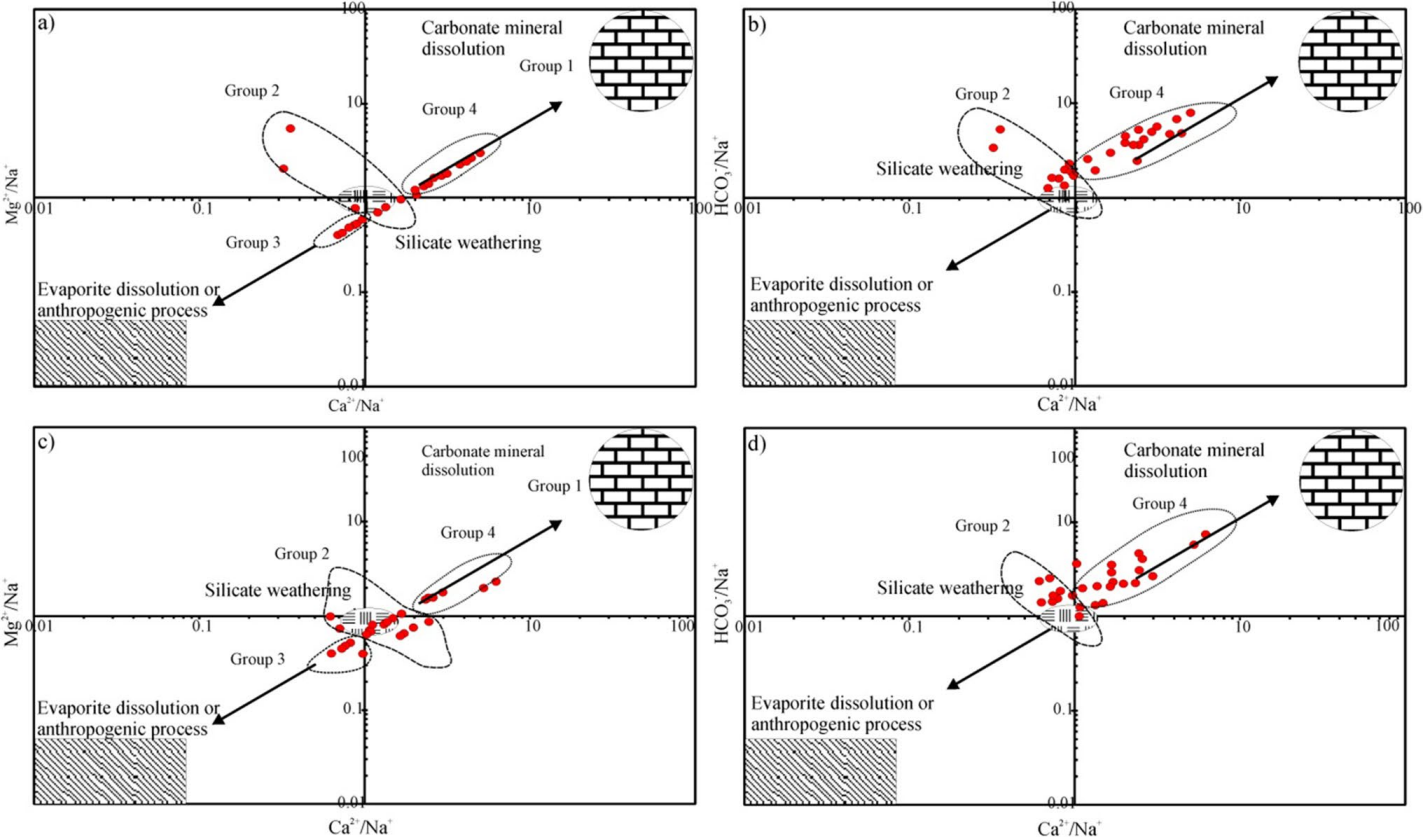
Plots of **a**, **c** Mg^2+^/Na^+^ versus Ca^+^/Na^+^ and **b**, **d** HCO_3_^−^/Na^+^ versus Ca^2+^/Na^+^ during the wet and dry season

In addition, the influence of feldspar weathering and CaCO_3_ dissolution also demonstrated Ca^2+^/Na^+^ against Mg^2+^/Na^+^ diagram (Fig. 6a, c) and Ca^2+^/Na^+^ against HCO_3_^−^/Na^+^ graph (Fig. 6b, d). The groundwater samples in the study area have been noted to fall within the regions associated with carbonate mineral/evaporation dissolution, and anthropogenic processes. During the wet season, a majority of groundwater samples are observed to fall within a molar ratio of group 4, suggesting that carbonate-silicate mixing predominantly influences the groundwater chemistry (Fig. 6a, b). However, during dry seasons, the majority of groundwater samples exhibit molar ratios falling within groups 4 and 2. This trend may be attributed to the decrease in groundwater levels reaching the minimum level during this period (Fig. 6c, d). Examples of the groundwater, especially located in or around urban regions, are in group 3 and show anthropogenic processes and silicate mixture. On the other hand, groundwater samples in group 2 indicate another source is silicate weathering (Marghade 2020).

### Human or anthropogenic inputs

**Fig. 7:**
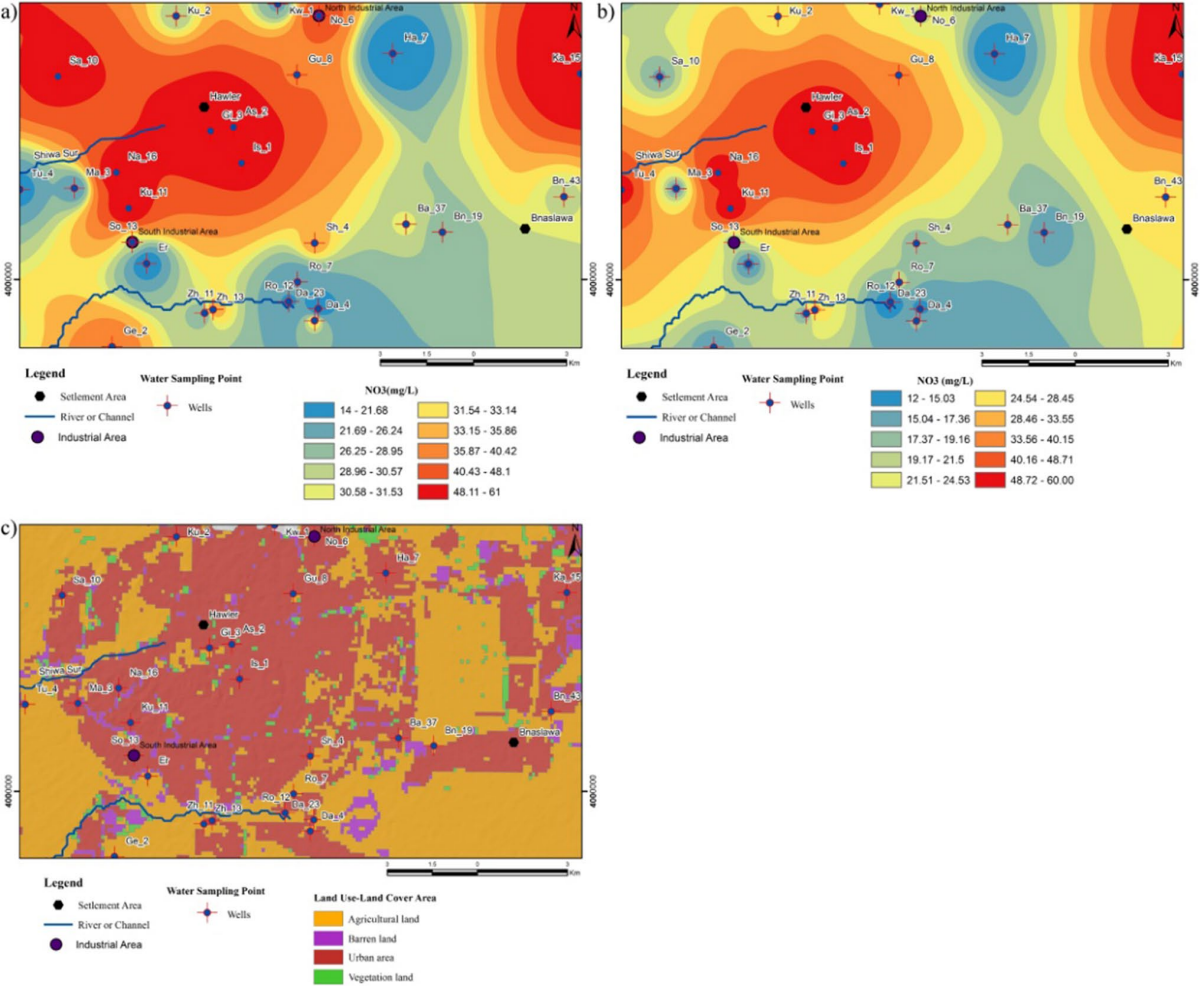
**a**, **b** Spatial distribution maps of the NO_3_^−^ in the well water samples during the wet and dry seasons and **c** land use/land cover map

The chemical properties of groundwater resources can be significantly influenced and, in some cases, driven by anthropogenic processes resulting from human activities together with natural processes. The exposition of variations in the hydrochemical composition of groundwater resulting from inputs of anthropogenic is a complex process containing uncertainties (Rao et al. 2017; Luo et al. 2021). Specifically, the NO_3_^−^ concentration of a body of water may indicate the impact of anthropogenic activities, including agricultural activities and domestic wastewater discharge, on both surface water and groundwater sources (Liu et al. 2021). In the study area, the concentration of NO_3_^−^ found in the groundwater samples varied between 14.00 and 61.00 mg/L with an average value of 35.70 mg/L during the wet season, whereas during the dry season, it was found to vary from 12.00 to 60.00 mg/L with an average value of 29.00 mg/L, respectively (Fig. 7a, b). High concentrations of NO_3_^−^ in groundwater (especially in dense urban areas) indicate that intensive human activities such as domestic wastes (cesspools), septic tank leakage, nitrification of organic N and NH_4_, and oxidation of organic substances, as well as excessive use of fertilizers in agricultural areas, are clearly visible from the land use/land cover (LULC) characteristics of the study area (Fig. 7c) (Tiwari and Singh 2014; Adimalla and Qian 2019; Egbueri et al. 2019; Smail and Dişli 2023). NO_3_^−^ concentration is higher than drinking water permissible limits of WHO (i.e., 50 mg/L) in 25.92% of the well water samples during the wet and dry seasons. The very high NO_3_^−^ concentrations exceeding 50 mg/L are detected in well numbers Ka-15, Sa-10, Na-16, Ku-11, Gl-3, As-2, and Is-1 during the wet season. Similarly, during the dry season, elevated NO_3_^−^ levels above 50 mg/L are observed in wells Ka-15, Tu-4, Na-16, Ku-11, Gl-3, As-2, and Is-1.

**Fig. 8:**
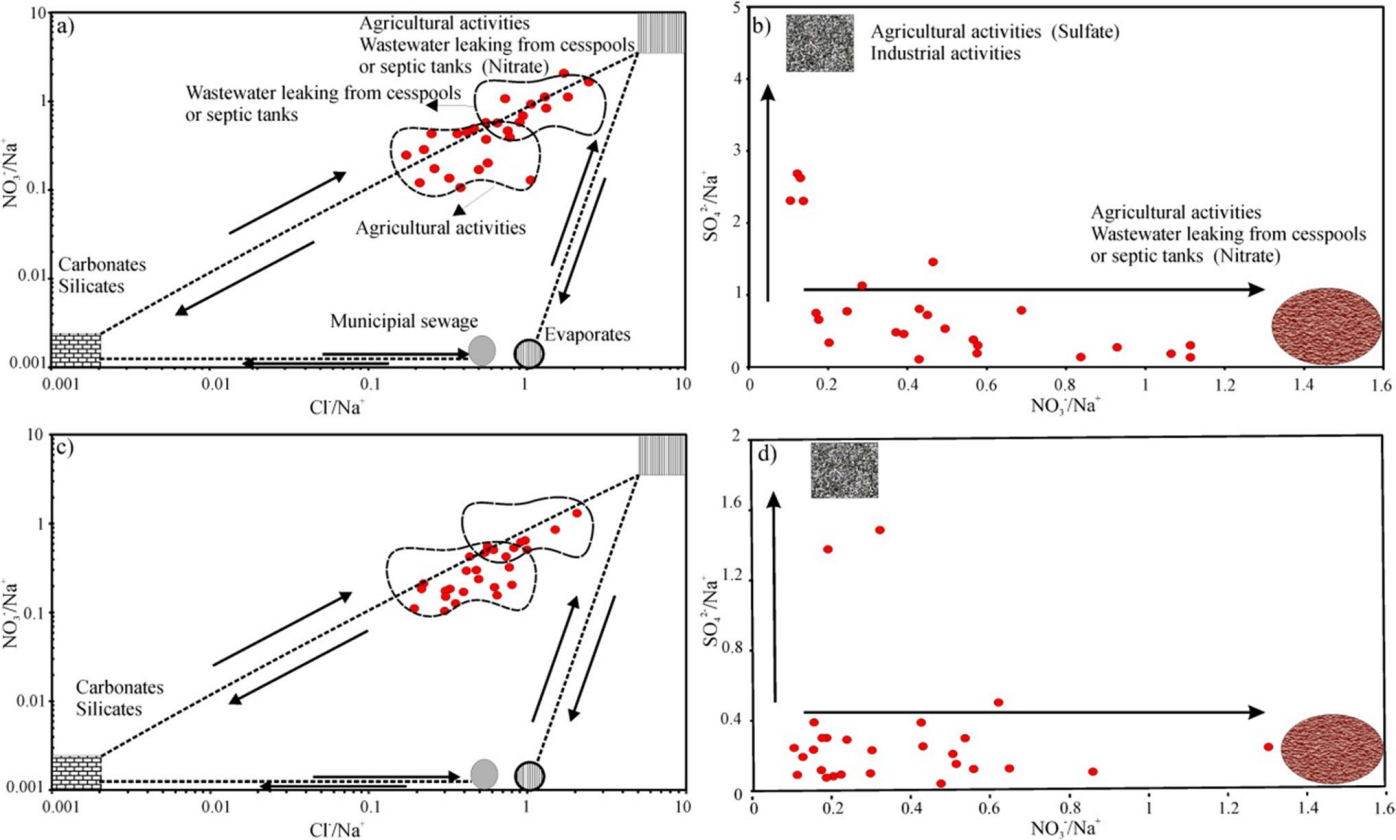
Plots of **a** Cl^−^/Na^+^ versus NO_3_^−^/Na^+^ and **b** NO_3_^−^/Na^+^ versus SO_4_^2−^/Na^+^ for groundwater samples during the wet and dry seasons

In contrast to natural (geological) processes such as atmospheric inputs and rock-water interaction, anthropogenic activities introduce industrial wastewater, agricultural fertilizers, and cesspools into the underground due to LULC variations. This results in the input of with intense human activities contributing to elevated levels of SO_4_^2−^, Cl^−^, and NO_3_^−^ (Xiao et al. 2017; Gao et al. 2022). Cl and SO_4_^2−^ can generally derive from the dissolution of evaporite rock like gypsum and halite, a different mechanism such as mineral oxidation containing sulfide and industrial activities, while NO_3_^−^ is mainly caused by human activity origins such as agricultural runoff and domestic sewage inputs (Liu et al. 2021). The plots of the Na^+^ normalized molar ratio analysis ((NO_3_^−^/Na^+^)-(Cl^−^/Na^+^)) are displayed in Fig. 8a, c as an indication of the effect of agricultural activities and wastewater leaking from cesspools or septic tanks on groundwater quality during the wet and dry seasons. Figure 7c illustrates that the majority of the groundwater sampling points containing high NO_3_^−^ concentrations (the content of NO_3_^−^ > 25) are predominantly situated near or within urban areas. The observed influence on dense settlements suggests that wastewater leakage from cesspools or septic tanks is the dominant contributor to the elevated levels of NO_3_^−^ in the groundwater sources. Furthermore, the high levels of NO_3_^−^ in certain samples can be attributed to intensive and long-term farming or agricultural activities in these regions, as depicted in Fig. 7c. This indicates that the groundwater system consisting of the Quaternary deposits and the Bakhtiari Formation in the Erbil Central Sub-basin is sensitive to the influence of point and non-point pollution. Figure 8a, c also indicate that there is a mixture of water effects of anthropogenic origin in well waters. Furthermore, as seen in Fig. 8b, d, the relationship between NO_3_^−^/Na^+^ ratios and SO_4_^2−^/Na^+^ ratios indicates that industrial activities have contributed to NO_3_^−^ in groundwater, at least slightly, compared to other anthropogenic processes. As seen in Fig. 8b, d, it was assumed that two different types of pollution during the wet and dry seasons were effective in polluting groundwater. The first of them consists of nitrate-containing fertilizers in agricultural areas and cesspool/septic tank systems, and the other is industrial wastewater (Gao et al. 2022).

**Fig. 9:**
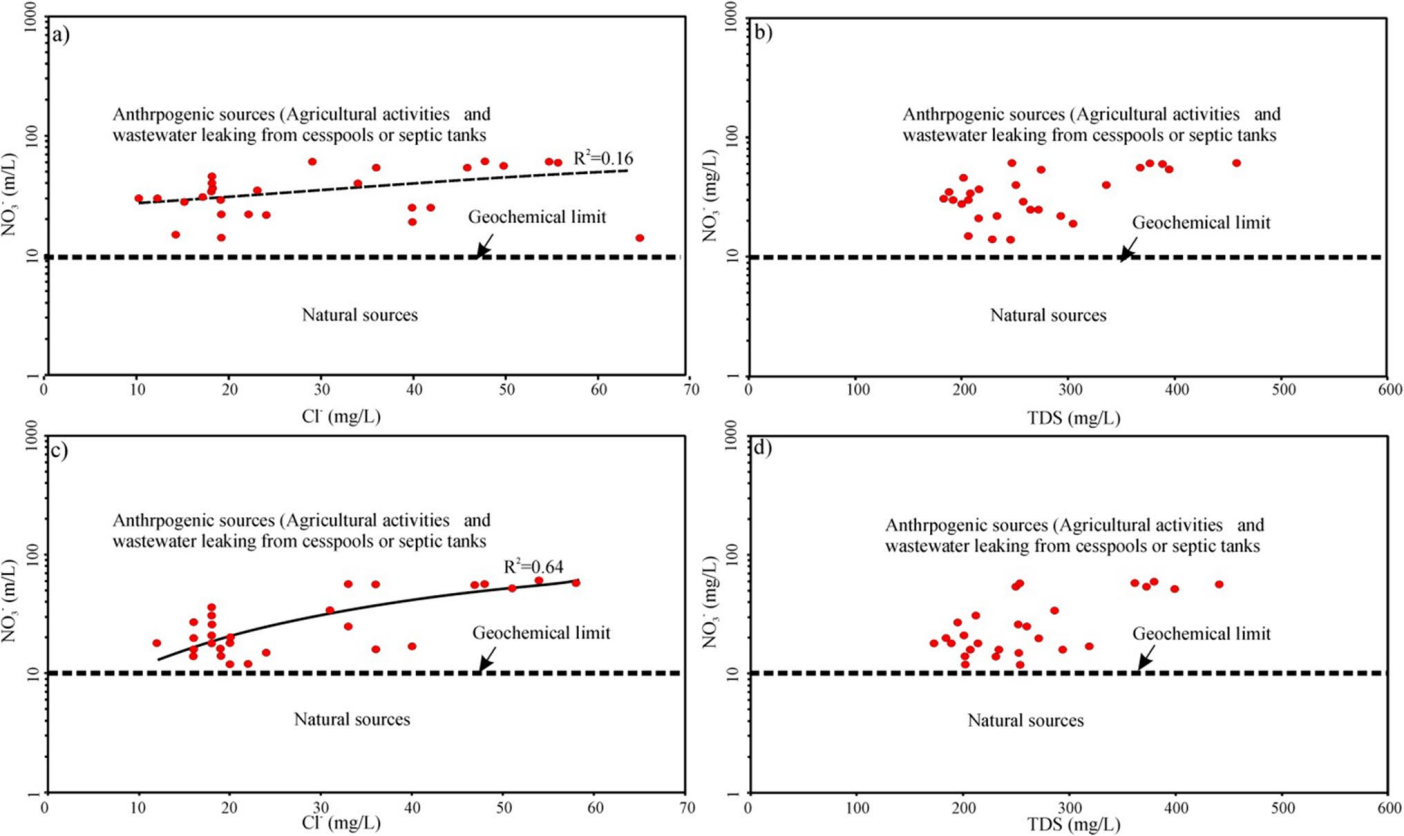
Scatter plot of **a**, **c** NO_3_^−^ versus Cl^−^ and **b**, **d** NO_3_^−^ versus TDS during the wet and dry seasons

Moreover, the nitrate contents in the well water samples have been noted to exhibit an increasing trend with the elevation of TDS and Cl^−^, as illustrated in Fig. 9. This observation suggests that the high nitrate content is likely a result of surface inputs of anthropogenic origin; they will simultaneously increase the concentration of nitrate and some other dissolved solids in groundwater (Xiao et al. 2022). As shown in Fig. 9a, c, the positive increasing relationship between NO_3_^−^ and Cl^−^ was observed with the correlation coefficient of 0.16 and 0.64 for the well waters during the wet and dry seasons; this indicates the role of agricultural activities and leaking from cesspools in controlling NO_3_^−^ concentration (Amiri et al. 2022). Furthermore, the observed similar relationship with the increasing trend of NO_3_^−^ content with TDS (Fig. 9b, d) supports all input of nitrates from anthropogenic activities (Li et al. 2016; Xiao et al. 2022).

### Groundwater quality assessment based on EWQI

We used EWQI method to evaluate the general groundwater quality for drinking purposes in the study area. Six health-interested groundwater quality parameters including pH, TH, BOD, COD, Ca^2+^, and NO_3_^−^ were concerned in the calculation of the EWQI and were determined as the most effective compounds in the varying groundwater composition in the study area (as per WHO limits). These six parameters were measured in eight different wells in the study area during wet and dry seasons. In this study area, during the wet season, the EWQI value of the groundwater samples was between 83.27 and 120.30 with an average value of 104.02, while in the dry season, the EWQI value ranged from 95.68 to 130.65 with an average value of 113.06 (Table 4). In the groundwater quality assessment (Table 4) during the wet season, the EWQI values reveal that approximately 62.5% of groundwater samples fall into the poor-quality water class (range IV), and are absolutely not recommended for drinking purposes. Additionally, 37.5% of the well water samples (Bn-19, Ka-15, Na-16) are classed as moderate-quality water (rank III) indicating that they are marginally suitable for drinking purposes. Similarly, in the dry season, approximately 75% of the water samples are categorized as poor-quality water (rank IV) while 25% fall into the moderate-quality water index (rank III). Consequently, the higher concentrations of BOD, COD, and NO_3_^−^ during the dry season compared to the wet season are parameters that significantly affect the results of EWQI.

**Table 4:**
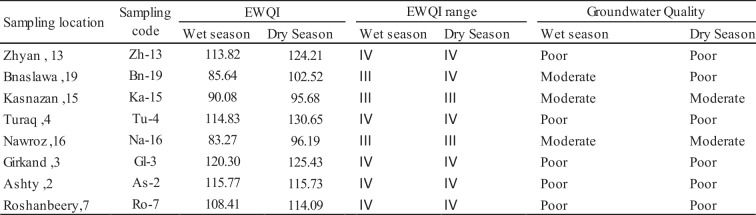
Classification of groundwater quality in the study area based on EWQI

**Fig. 10:**
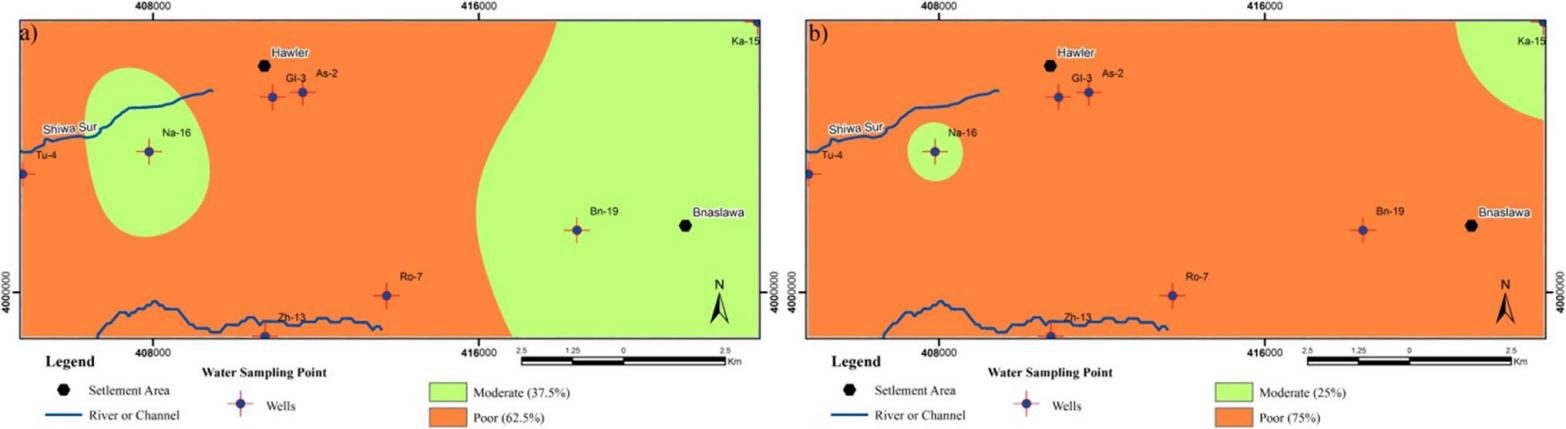
Classified spatial distribution groundwater quality based on EWQI in the wet season (**a**) and dry season (**b**)

The spatial mapping of the EWQI value classifications of water quality for groundwater samples collected from eight well samples in the study area is shown in Fig. 10a, b, was generated using the inverse distance weighting method in ArcGIS 10.2. In the wet season compared to the dry season, the predominant category of water quality covering the largest area is moderate water. As seen in Fig. 10a, groundwater samples with the quality of rank III (moderate quality) in the wet season are mainly concentrated around the western parts of the Hawler (Erbil) urban area (especially in well no. Na-16) and eastern part (between Bn-19 and Ka-15). Consequently, the groundwater quality in these regions is moderate, suggesting that it is generally suitable for drinking purposes on the whole. However, during the dry season, groundwater quality is generally represented by a high value of EWQI in Na-16 and Ka-15 wells indicating that the overall groundwater quality is poor and deemed not suitable for drinking (Fig. 10b). It has been determined that the contaminated areas are affected by seepage from cesspool points throughout the urban area and excessive use of fertilizers in agricultural lands, which can have a negative influence on the groundwater quality in the study area. In addition, it causes an increase in nitrate leaching through the “vadose zones” depending on the groundwater level, which generally increases at the end of the wet season and thus an increase in the nitrate concentration in the groundwater. The locations where poor groundwater quality in both seasons is concentrated mainly in settlement areas and agricultural lands (Fig. 10).

### Potential health risk assessment

Nitrate is a good index of groundwater quality and sensitivity, particularly in settlements lacking infrastructure, especially in agricultural areas, and its high concentrations in groundwater can pose health risks to humans (Wang and Li 2022). Nitrate concentrations in groundwater, particularly in the central regions of the city, generally exceed the permissible limits set by the WHO (2011) standard for drinking purposes. This trend is observed in both the urban areas of Hawler (Erbil) and Bnaslawa city. According to the standard guidelines of the United States Environmental Protection Agency (USEPA), nitrate is considered a non-carcinogenic risk to human health (Adimalla et al. 2020). The analysis of water samples collected from wells in the study area indicates that the groundwater is contaminated with nitrate. Therefore, HQ values were calculated to assess the potential non-carcinogenic risk associated with the oral intake of nitrate via drinking groundwater in adults, children, and infants. Table 5 displays the parameters of the health risk assessment model presented in this study.

**Table 5:**
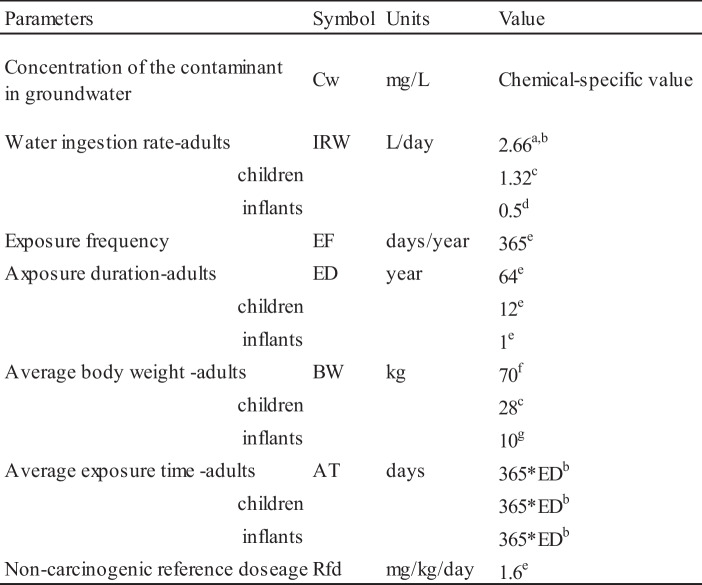
Values of parameters used in the health risk assessment for elevated nitrate in groundwater of the study area

^a^Reference to (USEPA 2008)

^b^Li et al. (2023)

^c^Gao et al. (2022)

^d^Liu et al. (2021)

^e^Adimalla et al. (2020)

^f^Sadeq and AbdulRahman (2019)

^g^Amiri et al. (2022)

The calculated results of the non-carcinogenic risks for adults, children, and infants due to the intake of drinking water containing high nitrate levels in groundwater resources are presented in Table 6 for both the wet and dry seasons. As demonstrated in Table 6, the calculated potential hazard index (HI) values for adults, children, and infants’ population for NO_3_^−^ varied in the range of 0.33–1.45, 0.41–1.80, and 0.44–1.91, respectively. The average values were 0.85, 1.05, and 1.12, respectively, during the wet season. However, during the dry season, HI-nitrate was in the range of 0.29–1.43 (average = 0.69) for adults, 0.35–1.77 (average = 0.85) for children, and 0.38–1.88 (average = 0.91) for infants, respectively**.** In majority of the well water samples, the HI values were found to be less than 1 (HI = 1) for adults, children, and infants during both seasons.

**Table 6:**
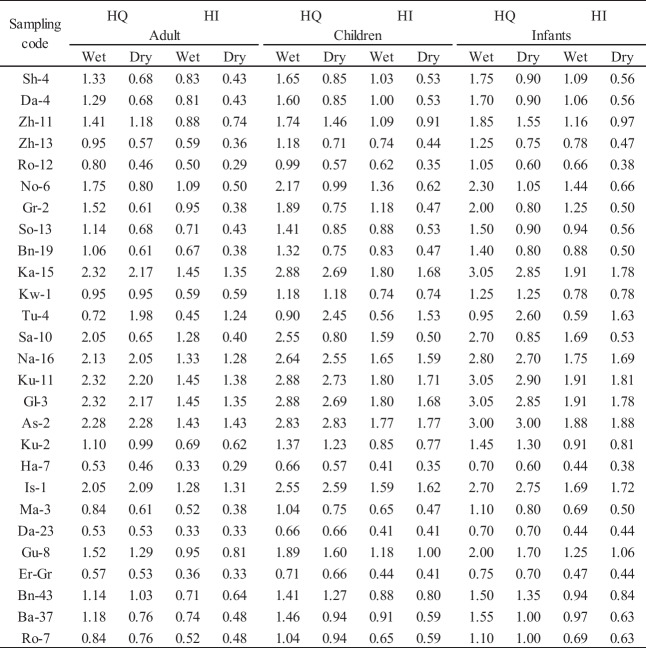
Result of health risk assessment through drinking water intake during the wet and dry seasons

The results of adult health risks revealed that during the wet and dry seasons, 29.6% and 25.9% of well water samples exceeded the permissible limit of non-carcinogenic risk (HI = 1.0). For children, the corresponding percentages were 48% and 30%, while for infants, they were 48.1% and 29.6%, respectively. These results suggest that adults are exposed to lower health risks in both seasons than children and infants. Furthermore, in terms of HI values, NO_3_^−^ indicates that it leads to the maximum non-carcinogenic health risk for children and infants, especially during the wet season.

**Fig. 11:**
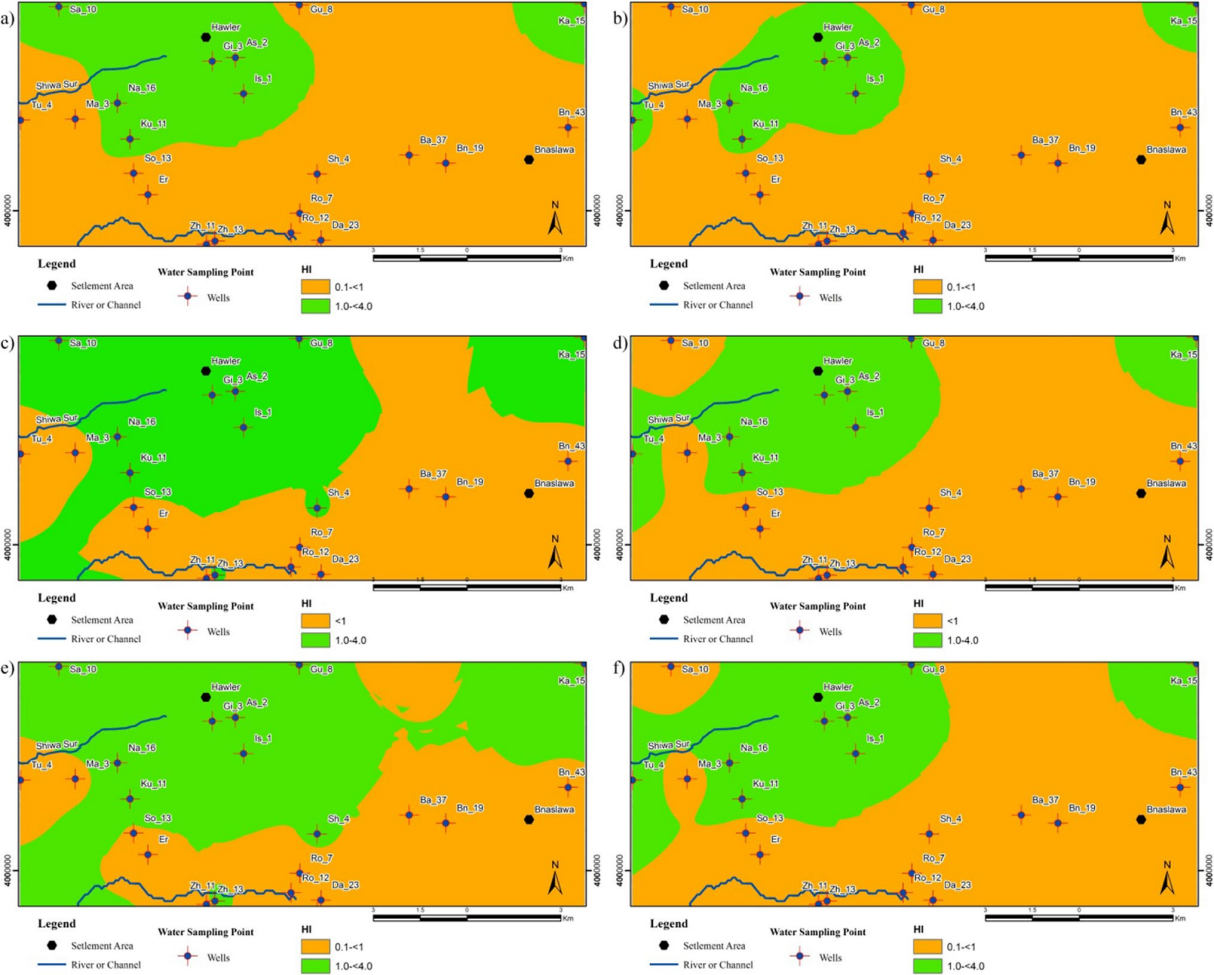
Spatial distribution maps of HI: **a**, **b** adults, **c**, **d** children, and **e**, **f** infants during the wet and dry season

Additionally, the implications of potential non-carcinogenic health risks are depicted in Fig. 11. The results reveal that the spatial distribution of health risks for infants, children, and adults is overall very similar and also indicate that the risk areas are predominantly concentrated in the north-northwest regions and the Hawler (Erbil) urban area. Figure 11 also shows that risks for adults were lower than children and infants, but higher for children than infants, across the study area during wet and dry periods. For children, areas with HI values exceeding 1 during the wet season expanded to include the Hawler region in southern, eastern, and western directions, while for infants, areas with HI values greater than 1 only covered the Hawler urban (Fig. 11c, e). The regions with the highest risks in both seasons have been observed near intensive human activities such as domestic wastes and septic tank or cesspool leakage as well as excessive use of chemical fertilizers in agricultural production poses another threat to human health in the study area. The southern and eastern parts of the study area have fewer settlements and more agricultural land, while the northern and northwestern parts have more residents and a higher population intensity. Therefore, the high risk, in the region, especially in the north and northwest, where the city of Hawler is located, indicates that it is also linked to human activities. Non-carcinogenic hazards to infants and children generally have the highest health risks relative to adults. This heightened vulnerability is attributed to their weaker resilience, less-developed enzymatic metabolism, and higher consumption per unit of body weight, making them more susceptible to nitrate poisoning (Duvva et al. 2021; Zhang et al. 2021; Sarma and Singh 2023).

## Conclusion

In this study, water quality data from the two different seasons, namely the wet and dry seasons, in May (2020) and September (2020) were selected. The hydrochemical characteristics, formation mechanisms, and quality of groundwater were evaluated using hydrochemical methods, GIS, and LULC. The sources of nitrate contaminant identified driving forces and were then employed to assess associated human health risks in the groundwater. In the well water samples, the groundwater exhibited alkali water quality, pH ranging from 7.10 to 8.00 and 7.10 to 7.90 during the wet season and 7.10 to 7.90 during the dry season. NO_3_^−^ concentration is higher than drinking water permissible limits of WHO (50 mg/L) in 25.92% of the groundwater samples during the wet and dry seasons. The order of abundance of the major ions in the groundwater samples is as follows: Ca^2+^ > Mg^2+^ > Na^+^ > K^+^ and Ca^2+^ > Na^+^ > Mg^2+^ > K^+^ for cations, and HCO_3_^−^ > NO_3_^−^ > SO_4_^2−^ > Cl and HCO_3_^−^ > SO_4_^2−^ > NO_3_^−^ > Cl^−^ for anions during the wet and dry seasons. The Na^+^/Cl^−^ molar ratio at 25.93 and 7.41% of the well water samples during the wet and dry periods is lower than 1. This indicates the influence of anthropogenic input or inverse ion exchange on groundwater. The plots of SO_4_^2−^ versus Ca^2+^, Ca^2+^ + Mg^2+^ versus HCO_3_^−^ + SO_4_^2−^, and Ca^2+^ versus HCO_3_^−^ demonstrate that dissolution of carbonate minerals is the dominant process while silicate mineral weathering occurs as a secondary predominant process in the groundwater system. The relationship between NO_3_^−^/Na^+^ versus Cl^−^/Na^+^ and NO_3_^−^/Na^+^ versus SO_4_^2−^/Na^+^ indicates that agricultural activities and wastewater leaking from cesspools or septic tanks play a significant role in impacting groundwater quality during both the wet and dry seasons.

The calculated EWQI values and spatial distribution maps reveal two vulnerable zones classified as moderate and poor groundwater quality during both the wet and dry seasons. High concentrations of NO_3_ and areas with poor groundwater quality in both seasons are concentrated in urban areas and agricultural lands, as seen in the LULC pattern. Nitrate contamination in the study area is primarily attributed to human activities, including the use of fertilizers, surface runoff, and leachates from cesspools or septic tanks in residential areas. The assessment of non-carcinogenic risk shows that the health risk in the wet season is higher than in the dry season, and the health risk of infants and children is much higher than adults. The non-carcinogenic risk level of the exposed population in the Hawler (Erbil) and Bnaslawa urban areas follows the order of descending risk as infants > children > adults. Additionally, the areas with a higher health risk (HI > 1) for both adults, children, and infants in both seasons are concentrated in the north-northwest of the study area, especially in the Hawler (Erbil) urban area. Elevated nitrate concentrations in groundwater cause a noticeable decrease in body mass, particularly in children and infants.

The important findings of this study can assist local governing bodies in the effective management of groundwater resources. Implementing awareness campaigns, enhancing the monitoring network, formulating effective policies, and fostering community cooperation are crucial steps for the sustainable development of groundwater reservoirs in this urban region.
